# The rise of astrocytes: are they guardians or troublemakers of the brain disorder?

**DOI:** 10.1038/s12276-025-01627-6

**Published:** 2026-02-05

**Authors:** Hee Yeon Kim, Seungchan Kim, Asli Nur Akaydin, Suhyun Kim, Seung Jae Hyeon, Junghee Lee, Hoon Ryu

**Affiliations:** 1 Brain Gene Regulation and Epigenetics Laboratory, Center for Brain Disorders, Brain Science Institute, Korea Institute of Science and Technology, Seoul, Republic of Korea; 2 Severance Hospital, Yonsei University College of Medicine, Seoul, Republic of Korea; 3 Boston University Alzheimer’s Disease Research Center and Department of Neurology, Boston University Chobanian & Avedisian School of Medicine, Boston, MA USA; 4 VA Boston Healthcare System, Boston, MA USA; 5 KHU-KIST Department of Converging Science and Technology, Kyung Hee University, Seoul, Republic of Korea

**Keywords:** Astrocyte, Alzheimer's disease

## Abstract

The brain is a highly complex, multicellular organ composed of diverse neuronal and nonneuronal cell types that function in concert to maintain central nervous system homeostasis. Among the glial populations, astrocytes are critical regulators of neuronal function. Under physiological conditions, astrocytes provide essential metabolic support, modulate neurotransmitter release and maintain neuronal health. Traditionally viewed as passive and supporting cells, astrocytes are now recognized as dynamic and responsive elements within the central nervous system. In response to pathological insults, astrocytes undergo significant changes in function, morphology and gene expression—a process known as reactive astrogliosis. Reactive astrocytes acquire heterogeneous characteristics that can contribute to brain disorders via the non-cell-autonomous mechanisms. However, the drivers of this transformation—and their shift from neuronal guardians to potential contributors to pathology—remain incompletely understood. Here we explore the complex, multidimensional roles of astrocytes and how reactive states alter their primary functions. We focus on the dual protective and pathological roles of astrocytes, particularly the transition from healthy to heterogeneous reactive forms, with the aim of understanding their overall impact on the progression of neurodegenerative diseases.

## Understanding astrocyte roles in brain health and disease

Astrocytes are star-shaped cells in the brain that support neurons by regulating their environment and providing nutrients. They can become ‘reactive’ in response to brain stress, such as in Alzheimer’s disease, where they may help or harm neurons. This study explores how astrocytes change during neurodegenerative diseases such as Alzheimer’s disease. Researchers examined astrocyte behavior using various methods, including cell cultures and animal models. They found that reactive astrocytes can either protect neurons by clearing harmful substances or contribute to disease progression by releasing toxic molecules. The study highlights the dual role of astrocytes: they can be both beneficial and detrimental depending on the context. Understanding these roles is crucial for developing treatments targeting astrocytes to slow or prevent neurodegeneration.

This summary was initially drafted using artificial intelligence, then revised and fact-checked by the author.

## Introduction

Astrocytes, a major type of glial cell in the central nervous system (CNS), play a pivotal role in neuronal survival and function. They regulate synaptic formation and function, release neurotrophic factors and neurotransmitters and maintain metabolic homeostasis in the brain. Although astrocytes support homeostatic salubrious functions to neurons in a healthy brain, they can also become toxic to neurons in a neurodegenerative brain^1^. It is now widely accepted that astrocytes can undergo morphological and molecular transformations, entering a ‘reactive’ state that may alter their function and disrupt the balance between their neuroprotective and neurotoxic properties. These cells may release proinflammatory cytokines and neurotoxic substances that exacerbate neuronal damage while also attempting to repair and maintain the tissue. The cellular and molecular mechanisms that determine the functional polarity of reactive astrocytes remain incompletely understood and are probably regulated by complex, context-dependent signaling networks^2,3^.

Astrocyte dysfunction is a common feature of neurodegenerative diseases such as Alzheimer’s disease (AD), Parkinson’s disease (PD) and amyotrophic lateral sclerosis (ALS). These conditions are characterized by the progressive neuronal degeneration and synaptic dysfunction, with astrocytes reactivity emerging as a key modulator of disease trajectory. During the progression of neurodegenerative disorders, astrocytes respond to chronic inflammation and pathogenic stimuli by acquiring distinct reactive phenotypes that act through non-cell-autonomous mechanisms to influence neuronal and glial homeostasis^4^. A major challenge in neurodegeneration research is to elucidate how the reactive astrocytes are regulated and how these phenotypic shifts contribute to disease progression.

In aging brains, astrocytes undergo structural and functional alterations. AD is an aging-associated brain disorder characterized by excessive amyloid-beta (Aβ) plaque deposition with the loss of cognitive and memory function. In AD, astrocytes not only take up and clear Aβ plaques but also contribute to disease progression by transforming into reactive astrocytes. These reactive astrocytes exhibit altered behaviors that can both support and disrupt neuronal function, creating a complex environment that accelerates neurodegeneration. The dual role of astrocytes in AD emphasizes the importance of understanding their transformation and interactions within the brain’s pathological landscape. With the increasing prevalence of AD due to rising life expectancy, basic and clinical studies have become more crucial to uncovering the mechanisms underlying its pathogenesis and developing effective treatment strategies, not only for AD but also for other brain disorders^5^. A deeper understanding of astrocyte reactivity will be pivotal for the development of astrocyte-targeted therapeutic strategies aimed at restoring CNS homeostasis and mitigating disease progression.

## Astrocytes: more than star-shaped glial cells in the brain

Astrocytes are nonneuronal cells in the brain and are among the most abundant cell types. They were first discovered by Rudolf Virchow in 1858 in his initial classification of glial cells^6^. Subsequent research by Camillo Golgi in the early 1870s revealed their heterogeneity, and the term ‘astrocyte’ was coined by Lenhossék to describe star-shaped cells in the CNS^7^. A major advancement in astrocyte research came with the identification of their reactive properties, facilitated by the discovery of glial fibrillary acidic protein (GFAP) as a reliable marker of astrocyte activation^4^.

Astrocytes are estimated to outnumber neurons by approximately fivefold in the CNS. Originating from ectoderm-derived neural stem cells in the neural tube, astrocyte lineage commitment begins with radial glia cells, which express classical markers such as GFAP, glutamate aspartate transporter (GLAST) and calcium-binding protein (S100B)^1^. As neurodevelopment progresses, radial glia cells differentiate into astrocytes, which migrate and diversify across brain regions into phenotypically and functionally distinct subtypes^8^.

**Fig. 1:**
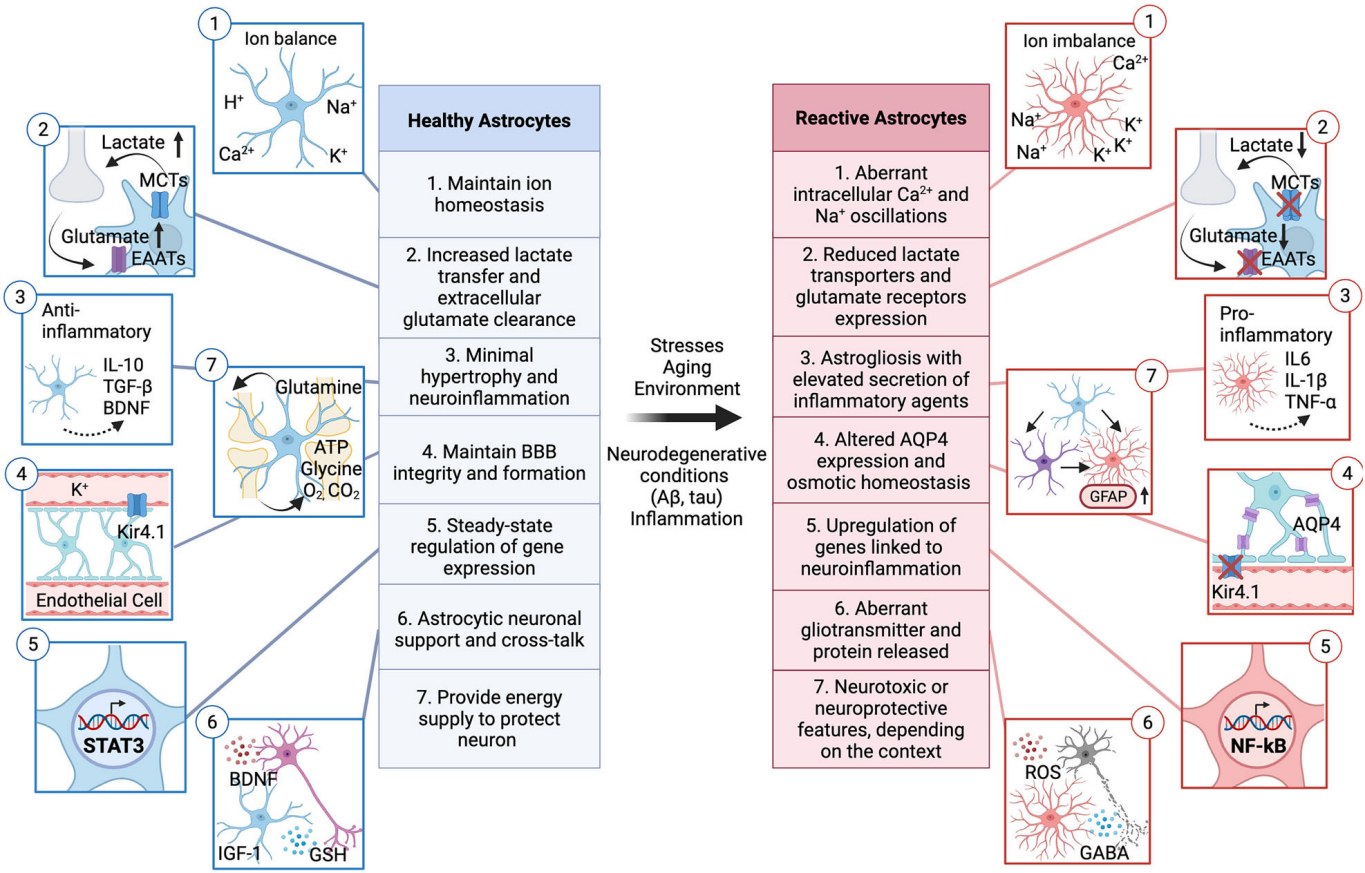
Astrocytes are involved in diverse regulatory functions within the brain. Healthy astrocytes (left, blue) support and protect neurons, whereas reactive astrocytes (right, red) alter their functions with heterogeneous characteristics linked to different reactive astrocyte states. It is important to note that not all functions are carried out by a single astrocyte, and not all physiological functions are lost during the reactive state.

The two main subtypes, classified by morphology and region, are protoplasmic and fibrous astrocytes. Protoplasmic astrocytes are located in gray matter and exhibit a highly branched, star-shaped morphology, where they commonly contribute to tripartite synapses. By contrast, fibrous astrocytes are predominantly found in white matter and display a fiber-like structure. Although fibrous astrocytes are less frequently associated with classical tripartite synapses, they modulate axonal conduction and participate in axo–glial interactions that share functional similarities with tripartite arrangements, thereby supporting neuronal communication and structural integrity. With their diverse array of pumps and transporters, astrocytes are capable of delivering beneficial substances, including ions, neurotransmitters and metabolites, while also producing or releasing detrimental molecules, such as reactive oxygen and nitrogen species. Astrocytes have diverse roles in the maintenance of healthy CNS as shown in Fig. 1; they take up or degrade neurotransmitters, regulate synaptic connectivity, release various factors with gland-like properties and even contribute to high-order cognitive functions through their integration within neuronal circuits. Astrocytes also modulate the structure and function of the blood–brain barrier (BBB), a role that has evolutionary roots in glial-controlled vascular regulation observed in primitive vertebrates, with endothelial cell dominance emerging in higher speices^9,10^.

Although the beneficial roles of astrocytes in the maintenance of neuronal function have long been recognized, recent advancements in research have begun to alter the perspective regarding their function and role in the brain, suggesting that their contributions should be examined at the cellular level^11^. As emphasized by Zhao et al., the prolonged progression of AD before clinical symptoms emerge indicates that astrocytes may substantially contribute to disease pathogenesis at a single-cell resolution^12^. This underscores the necessity of investigating astrocytes beyond bulk tissue analyses, as single-cell studies reveal that astrocyte subpopulations exhibit heterogeneous responses to pathological stimuli. Astrocytes actively influence pathogenesis through functional receptors and the release of signaling molecules. In response to oxidative agents, inflammation, infections or neurodegeneration, astrocytes undergo profound changes in morphology, gene expression and function—a phenomenon known as astrocyte state transition. Notably, chronic stress can trigger epigenetic modifications, reprogramming astrocytes into disease-associated reactive states with distinct molecular profiles compared with normal astrocytes^9,11^. Single-cell RNA sequencing (RNA-seq) has demonstrated that reactive astrocytes exhibit transcriptional diversity, with specific subsets adopting either neuroprotective or neurotoxic phenotypes. Furthermore, a recent study has highlighted astrocyte-mediated epigenetic regulation as a key factor to affect autophagy in AD, implicating altered gene expression as a driver of disease pathology^13^. Thus, understanding transcriptomic and epigenetic regulation in astrocytes could clarify their precise role in neurodegenerative diseases and offer a potential pathway for leveraging their intrinsic heterogeneity to promote neuroprotection and counteract disease progression (Table 1).

**Table 1:** AD-associated astrocyte phenotypes across models, regions, stages and methods.

Study model	Disease stage	Brain region	Key astrocyte findings	Method	Reference
Mouse (APP/PS1, 5xFAD)	Mid–late AD	Hippocampus (CA1/CA3/DG)	Reactive astrocytes produced excessive GABA via MAO-Band BEST1 channels, which impaired synaptic plasticity and memory	IHC, electrophysiology	Jo et al.^58^, *Nature Medicine*
Mouse (APP/PS1)	AD stage	Hippocampus, cortex	Treatment with galantamine reduced Aβ deposition and astrocyte activation, thereby improving cognitive performance in APP/PS1 mice	IHC, ELISA	Wu et al.^151^, *Experimental Gerontology*
Mouse (APP/PS1)	Early	Hippocampus	TRPA1 activation in astrocytes led to calcium hyperactivity and consequent synaptic dysfunction in response to Aβ exposure	Electrophysiology, IHC, WB	Bosson et al.^154^, *Molecular Neurodegeneration*
Human (patient with AD plasma exosome)	Clinical AD	Systemic	Astrocyte-derived exosome was enriched with complement and inflammatory proteins, promoting neuroinflammation in patients with AD	Cerebrospinal fluid/plasma exosome assay	Goetzl et al.^56^, *Annals of Neurology*
Immortalized astrocytes from human APOE targeted-replacement mice (E2/E3/E4), in vitro	–	–	APOE4 astrocytes showed reduced glucose uptake and altered energy metabolism, with enhanced lactate production and tricarboxylic acid cycle remodeling	Metabolomics	Williams et al.^81^, *Neurobiology of Disease*
Human postmortem, mouse (3xTg, APP/PS1, Tg2576, arcAβ, 5xFAD, ICV-STZ)	Early–late AD	Hippocampus, cortex	Downregulation of astrocytic GLUT1 contributed to cerebral glucose hypometabolism in AD	IHC, WB, liquid chromatography–tandem mass spectrometry and dynamic fluorodeoxyglucose-positron emission tomography	Kyrtata et al.^75^, *Frontiers in Neuroscience*
Human postmortem, mouse (APP/PS1), human astrocytes	Late AD	Cortex	Upregulation of NOX4 in astrocytes induced lipid peroxidation and mitochondrial impairment, driving ferroptosis in the AD brain	WB, IF, biochemical assays, ROS imaging	Park et al. ^157^, *Redox Biology*
Mouse (APP/PS1)	AD stage	Hippocampus	Activation of the astrocytic urea cycle detoxified Aβ-derived ammonia but simultaneously increased tonic GABA release, leading to memory impairment	RNA-seq, metabolomics	Ju et al.^59^, *Cell Metabolism*
Mouse (APP/PS1)	Early–mid AD	Hippocampus, cortex	Astrocytes in AD mice exhibited impaired Ca^2+^ signaling and network activity, correlating with neuronal dysfunction and behavioral deficits	Two-photon, IHC, transcriptomics	Åbjørsbråten^114^, *eLife*
Human astrocytes, in vitro, mouse (5xFAD; BACE1 floxed, GFAP-cre)	Early AD	Hippocampus	Upregulation of BACE1 in astrocytes facilitated Aβ accumulation in vitro	RNA-seq, biochemical assays, IHC, WB	Zhou et al.^54^, *Molecular Neurodegeneration*
Human postmortem	Clinical AD	Cortex	Reactive astrocytes in patients with AD exhibited a loss of homeostatic gene expression profiles compared with controls	Single-nuclei RNA-seq	Dai et al.^27^, *Acta Neuropathologica Communications*
Mouse (APP/PS1), primary astrocytes, human patient brain tissue	AD stage	Hippocampus, cortex	Induction of autophagy markers LC3B and SQSTM1 in astrocytes enhanced Aβ clearance, whereas inhibition of autophagy aggravated oxidative stress and cognitive decline	WB, IHC, RNA-seq	Kim et al.^13^, *Molecular Neurodegeneration*
Human AD samples iPS cell-derived astrocytes	Clinical AD	Cortex	Levels of PRDM16-DT IncRNA were reduced, whereas NEAT1 was increased in astrocytes; changes linked to cognitive impairment in AD	Single-nuclei RNA, IncRNA profiling	Schröder et al.^33^, *Acta Neuropathologica*
Human patient brain tissue, mouse (P301S; PS19)	AD stage	Hippocampus, cortex	Astrocytic HDAC7 deacetylated TFEB, suppressing lysosome biogenesis and thereby exacerbating tau pathology	RNA-seq, IHC	Ye et al.^29^, *Molecular Neurodegeneration*

This table consolidates studies cited in the review astrocytic alterations in AD. For each study we list the model (human, mouse), AD stage, brain region, methods and concise astrocyte-related findings spanning Aβ/tau pathology, synaptic/ion and metabolic remodeling, oxidative stress and BBB integrity. *IHC* immunohistochemistry; *WB* western blotting.

## Exploring the multifaceted functions of astrocytes in the brain

### Astrocytes are a jack-of-all trades in the brain

Astrocytes have many functional, physiological and metabolic roles in regulating the nervous system across different regions, including the brain, spinal cord and retina^11^. Their strategic positioning between blood vessels and synapses allows them to regulate neurovascular coupling and support neuronal acticity^14^. A single astrocyte domain can encompass approximately two million synapses, with their interaction potential further enhanced with gap junctions. Astrocytes can function as secretory cells, releasing a variety of signaling molecules—including classical neurotransmitters (ATP, GABA, glutamate and glycine), gliotransmitters (D-serine and glycine), metabolic substrates (lactate) and growth factors (BDNF and TGF-β)—through exocytic vesicles, transporters or channels. Although these secretions have been proposed to fine-tune synaptic transmission and support neuronal metabolism, the precise mechanisms and physiological relevance of gliotransmission remain a matter of active debate^15–17^.

Astrocytes are involved in the formation and regulation of the BBB, maintaining blood flow and osmotic balance. They secrete modulatory molecules, including prostaglandins, arachidonic acid and nitric oxide, which influence vessel diameter and blood flow in response to neuronal activity^8,18^. In addition, astrocytes form local signaling networks with other neurons and their surrounding environment. Ionotropic receptors such as α-amino-3-hydroxy-5-methyl-isoxazole propionate (AMPA), *N*-methyl-D-aspartate (NMDA) types of tetrameric glutamate receptor (GluR) and P2X trimeric purinoceptors enhance synaptic transmission by accelerating vesicular transport. Astrocytes regulate cellular osmotic balance and extracellular ion homeostasis by mediating water and potassium (K^+^) transfer. This is primarily mediated by aquaporin water channels (for example, AQP4) and inwardly rectifying K^+^ channels such as Kir4.1, which are often colocalized at astrocytic endfeet adjacent to blood vessels. Their importance in brain osmoregulation and tissue swelling has been demonstrated in global AQP4 knockout mouse models. Moreover, astrocytes trigger neurogenesis by releasing neurogenesis-related factors such as Wnt3, insulin-like growth factor binding protein 6, interleukin 1β and interleukin 6. They have roles in gray and white matter formation as they lead the way for neuroblasts and migrating axons^9,19^. Through connexin gap junctions, astrocytes facilitate the exchange of Ca^2+^ and K^+^ ions and signal molecules, functioning as ion buffers. They also participate in the metabolic conversion of glutamate to glutamine and store glycogen, which can be utilized during high cognitive activity and hypoglycemia state. Furthermore, astrocytes produce lactate from glucose and ketones, providing an alternative energy source for neurons^20^.

## Astrocyte state transitions: balancing protection and pathology

Traditionally, neuronal plasticity has been regarded as the principal mechanism underlying adaptive responses in the CNS, encompassing synaptic remodeling and circuit reorganization that support learning and memory. Yet, emerging evidence highlights that astrocytes also undergo dynamic state transitions, adjusting their morphology, signaling and gene expression in ways that influence neuronal and network function. These transitions are context-dependent and potentially reversible, representing how astrocytes shift between distinct molecular and functional profiles in response to physiological or pathological stimuli. The diversity arising from such transitions contributes to the reactive heterogeneity observed among astrocyte populations across disease contexts. Through these dynamic changes, astrocytes reshape their interactions with synapses, blood vessels and immune cells—manifested through changes in calcium (Ca²⁺) signaling, neurotransmitter uptake, metabolic support and secretion of gliotransmitters and cytokines. Collectively, astrocytic state transitions and the resulting heterogeneity enables glial networks to flexibly sustain neuronal activity and preserve circuit homeostasis. In the following sections, we delineate the mechanisms and regulatory dimensions governing these astrocytic state transitions and their contributions to reactive diversity under environmental stressors and pathological challenges.

### Astrocytic autophagy plasticity

**Fig. 2:**
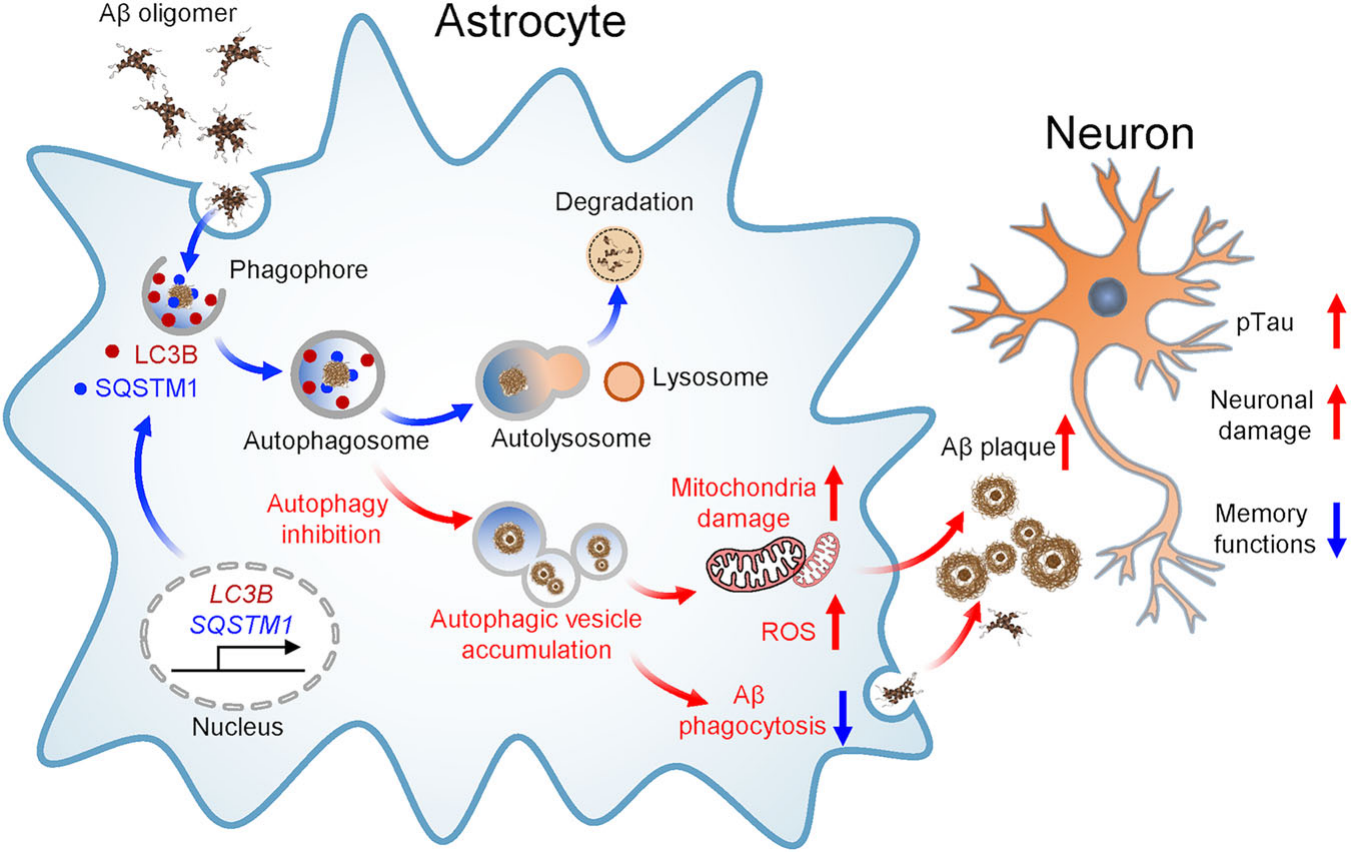
Astrocyte autophagic plasticity modulate Aβ clearance in AD. Astrocytes exhibit distinct responses to external stimuli depending on the duration and severity of the insult. Astrocytes exhibit an autophagy plasticity, a novel mechanism, in that they induce *MAP1LC3B/LC3B* and *SQSTM1* genes in response to AD stresses such as Aβ oligomers. The sequential induction of autophagy-related genes in astrocytes contributes to the clearance of Aβ in the brain of AD. Blue arrows indicate the normal status of the autophagy pathway in response to Aβ oligomers, whereas red arrows indicate the disease state of the autophagy pathway. The scheme is modified from *Molecular Neurodegeneration* (Kim et al.^13^).

Among the diverse dimensions of astrocyte state transitions, autophagy represents a key regulatory axis that allows astrocytes to adapt to metabolic and proteotoxic stress. Through the fine-tuned modulation of autophagic flux, astrocytes maintain cellular homeostasis and contribute to neuronal protection under disease conditions. This section highlights how the dynamic regulation of the autophagy machinery underlies astrocytic adaptability in response to pathological stressors such as Aβ accumulation. Recent evidence, including our own findings, demonstrates that autophagy components are induced by Aβ oligomers in astrocytes^13^. In particular, LC3B and SQSTM1, two major autophagy regulators, are significantly upregulated alongside increased GFAP expression. By contrast, neurons exposed to Aβ oligomers show the downregulation of autophagy components in parallel with reduced MAP2 levels. These findings indicate that, under AD conditions, astrocytes and neurons exhibit distinct autophagic responses. Astrocytes react to Aβ-induced stress by dynamically modulating their autophagy machinery to enhance the clearance of Aβ oligomers. Aβ oligomers transiently elevate the expression of the *MAP1LC3B/LC3B* gene while sustaining the prolonged transcription of it, a pattern also observed in response to proinflammatory stimuli such as LPS and TNF-α. This temporal regulation of autophagy genes promotes Aβ degradation through the activation of astrocytic autophagy, whereas the pharmacological inhibition of autophagy exacerbates mitochondrial dysfunction and oxidative stress, ultimately leading to astrocyte death. The impairment of astrocytic autophagy plasticity due to the loss of LC3B and SQSTM1 function accelerates Aβ aggregation and increases GFAP-positive astrocytes in AD mice, thereby amplifying neuronal damage and memory deficits^13^ (Fig. 2). Beyond Aβ clearance, astrocytes also display resilience to proteotoxic stress by activating protective pathways, including glutathione synthesis and heat shock protein expression. For instance, following sublethal MG132 exposure, astrocytes acquire adaptive resistance to subsequent insults, highlighting a stress-induced plasticity that safeguards neuronal integrity in degenerative environments^21^.

### Astrocytic morphological state transitions

Astrocytes dynamically extend perivascular processes to establish contact with endothelial cells, forming and maintaining the neurovascular unit during development. This morphological state transition is critical for regulating BBB permeability and facilitating neurovascular coupling in response to local neural activity^22^. Moreover, actin and tubulin cytoskeletons in astrocytes exhibit dynamic reorganization during polarized migration, a process that correlates with localized changes in membrane stiffness and cellular motility. Stimulated emission depletion microscopy and atomic force microscopy-based analysis revealed that this cytoskeletal adaptability facilitates the spatial compartmentalization and mechanical responsiveness of astrocytes to environmental stimuli^23^. Astrocytes also show heterogeneity by reflecting inflammatory and metabolic changes. Astrocytic processes undergo rapid and reversible morphological remodeling in response to metabolic signals such as fasting and high-fat diet, a process regulated by IKKβ–nuclear factor kappa-light-chain-enhancer of activated B (NF-κB) signaling. The chronic activation of this inflammatory pathway compromises astrocyte process adaptability, leading to disrupted metabolic homeostasis manifested as glucose intolerance and hypertension^24^.

### Astrocytic epigenetic state transitions

Astrocytes exhibit functional changes, partly regulated by epigenetic reprogramming in the pathogenesis of AD. Epigenetic alterations in astrocytes including DNA methylation, histone modifications and noncoding RNA (ncRNA) activity are modulated by environmental and pathological factors such as Aβ exposure and inflammation. A global reduction in 5-methylcytosine (5mC) levels, indicative of widespread hypomethylation, has been reported in astrocytes of postmortem AD brains, potentially leading to aberrant gene activation^25^. In general, the hypermethylation of specific gene regions can silence protective genes, whereas hypomethylation may activate pathogenic pathways^26^. For example, GFAP expression is influenced by epigenetic regulation, probably involving changes in DNA methylation^27^. The DNA demethylase ten-eleven translocation (TET1) plays a central role in this process, and its mutations are associated with increased risk for early onset AD. Loss-of-function of Tet1 in AD mouse models exacerbates Aβ plaque accumulation. Under physiological conditions, TET enzymes mitigate AD-related pathology by reducing Aβ aggregation and tau hyperphosphorylation^28^. The dynamic balance between DNA methyltransferases and TET enzymes functions as a critical regulatory axis, shifting astrocyte gene expression profiles from homeostatic maintenance toward neuroinflammation and neurodegeneration-promoting states.

Histone modifications provide an additional layer of epigenetic regulation in astrocytes. These modifications alter chromatin accessibility and transcriptional activity^29^. Histone deacetylases (HDACs), in particular, have emerged as key regulators of astrocyte-mediated neuroinflammation and neurodegeneration. The inhibition of specific HDAC isoforms has shown therapeutic promise in AD models, improving cognitive function and reducing pathological hallmarks^30^. HDAC3 and HDAC11, for instance, regulate oxidative stress responses and Aβ metabolism, whereas HDAC7 contributes to tau pathology by suppressing lysosomal biogenesis through transcription factor EB (TFEB) deacetylation and by enhancing inflammatory signaling via the IKK–NF-κB pathway^31^. Furthermore, disease-associated changes in active histone marks such as H3K27ac and H3K4me3 correlate with transcriptional shifts in reactive astrocytes, particularly in genes associated with synaptic dysfunction, tau pathology and Aβ processing. These findings suggest isoform-selective HDAC inhibitors could provide more precise and safer therapeutic strategies compared with broad-spectrum HDAC blockade.

ncRNAs, including microRNAs (miRNAs) and long ncRNAs (lncRNAs), also exert significant posttranscriptional control over astrocyte gene expression^32^. In AD, dysregulated miRNAs such as miR-146a, miR-155, miR125b and miR-223-3p modulate key neuroinflammatory pathways. Alongside this, lncRNAs are involved in maintaining astrocytic support of neuronal and synaptic function. For example, PRDM16 divergent transcript (PRDM16-DT) is downregulated in AD, whereas nuclear enriched abundant transcript 1 (NEAT1) is upregulated and associated with reactive astrocyte states and cognitive impairment^33^. Astrocyte-specific or enriched ncRNAs act as finely tuned molecular switches that determine astrocyte phenotypes and regulated process such as NF-κB signaling, NLRP3 inflammasome activation and synaptic homeostasis. Collectively, these diverse epigenetic modifications orchestrate complex gene expression changes that governs astrocyte state transition. They modulate neuroinflammation, neuronal viability, Aβ metabolism and synaptic function—key determinants of AD progression. Understanding astrocyte-specific epigenetic signatures thus represents new avenues for identifying novel biomarkers and developing precision therapeutics targeting glial dysfunction in AD.

### Indirect astrocyte state transitions: astrocytes are the modulator of neuronal plasticity

It is well established that astrocytes modulate neuronal plasticity indirectly by secreting synaptogenic and anti-synaptogenic cues. For instance, Hevin promotes excitatory synapse formation by bridging neurxin 1α and neuroligin 1, whereas SPARC antagonizes this effect, thereby refining synaptic connections. Through these actions, astrocyte-derived molecules orchestrate both Hebbian and homeostatic plasticity, ensuing balanced neural circuit remolding throughout development and adulthood^34^.

## Reactive astrocyte: what happens when astrocytes face stresses and what are known reactive functions of astrocytes?

Astrocytes are highly responsive glial cells that interact with immune and neural cells and become activated when encountered with any foreign insult, including chronic injuries, infections, acute trauma and neurodegenerative diseases^20^. This reactive transformation entails morphological, functional and transcriptional remodeling, forming a continuum of activation states ranging from transient, protective responses to chronic, neurotoxic phenotypes. A refined definition of astrocyte reactivity includes four key features: (1) cellular, molecular and functional changes in response to CNS insults, (2) graded responses depending on insult severity, (3) context-dependent reactions influenced by intra- and intercellular signaling and (4) beneficial or detrimental effects on surrounding cells^35^.

Reactive astrocytes exist along a spectrum of activation, ranging from mild or moderate to severe and chronic states. In certain contexts, they can transition into severe reactive astrocytes, often characterized by proliferative changes and markers such as monoamine oxidase B (MAO-B) and GFAP upregulation. Under neurotoxic conditions, some astrocytes adopt maladaptive phenotypes—referred to as neurotoxic astrocytes—induced by microglial cytokines, characterized by the loss of supportive functions and secretion of factors that damage neurons. The activation of astrocytes exerts highly context-dependent functions that vary along the temporal course of disease, the nature of the pathological milieu and their heterogeneous transcriptional states. In experimental autoimmune encephalomyelitis, suppressing reactive astrocytes in established disease exacerbates severity by enhancing myeloid cell infiltration, highlighting a protective barrier function^36^. By contrast, Mayo et al. reported that astrocyte ablation in chronic experimental autoimmune encephalomyelitis ameliorates pathology, whereas acute-phase ablation worsens disease, illustrating a time-dependent dual role^37^. Similar context-dependent outcomes have been described in models of traumatic brain injury, where astrocytic reactivity initially limits tissue damage but later contributes to glial scar formation and synaptic dysfunction^38,39^. Transcriptional profiling studies further reveal that astrocyte subpopulations adopt divergent states ranging from neuroprotective to neurotoxic phenotypes in neurodegeneration, further underscoring the importance of astrocytic heterogeneity^40,41^. Collectively, these findings indicate that reactive astrocytes cannot be ascribed a uniform role; instead, their functions are shaped dynamically by temporal progression, pathological context and intrinsic state diversity^42^.

**Fig. 3:**
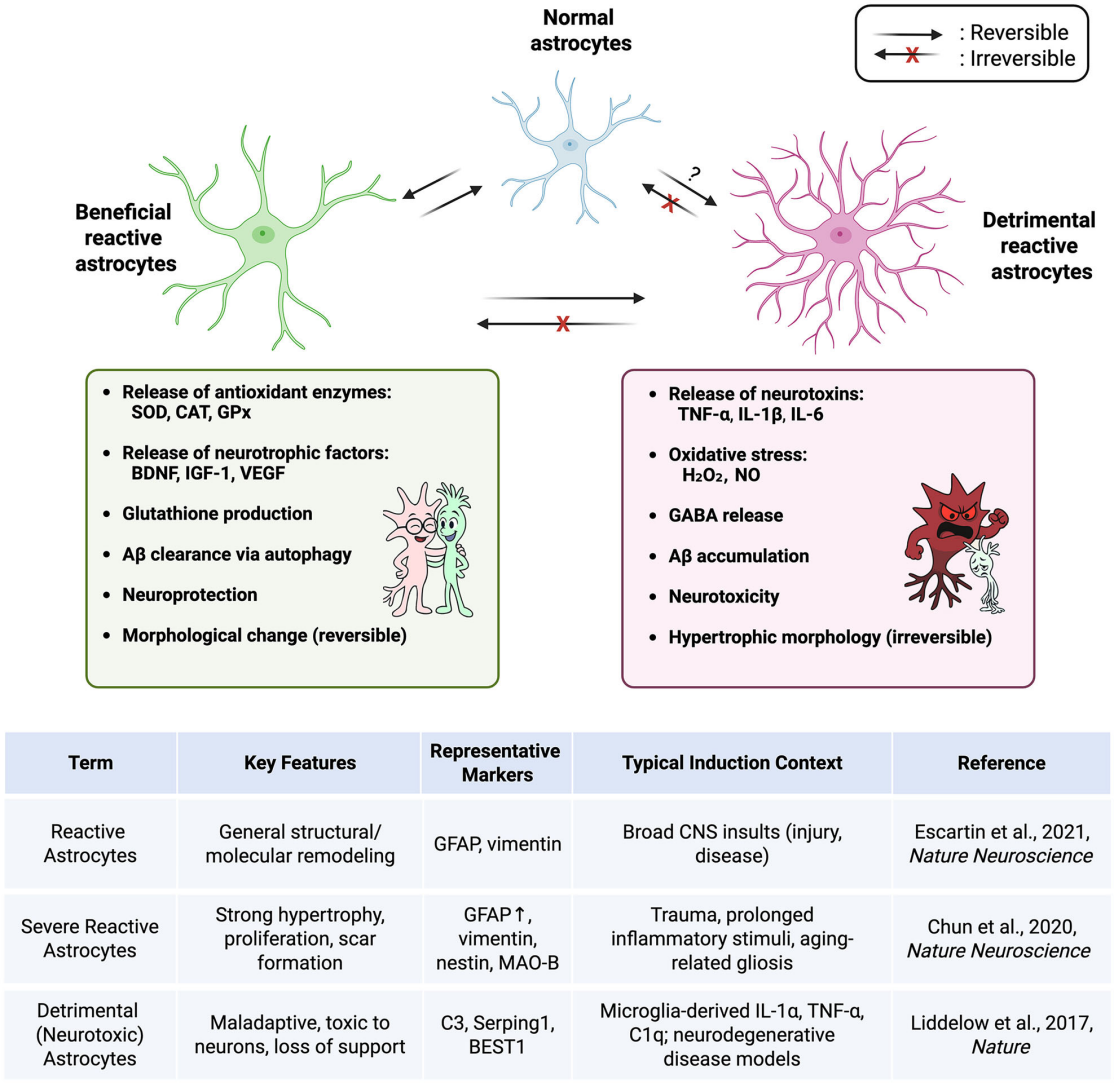
Astrocytes exhibit distinct responses to external stimuli depending on the duration and severity of the insult. Under mild or transient stress conditions, astrocytes become reactive and can resolve the situation in a neuroprotective manner and, in part, they can revert to the normal state. However, when stress level exceeds their threshold, reactive astrocytes undergo pathological transformation, acquiring toxic properties and exerting harmful effects on neighboring neuronal cells via the non-cell-autonomous pathways. These reactive astrocytes lose the capacity to return to their normal function. The precise epigenetic mechanism by which homeostatic astrocytes transition into a harmful reactive state remains unclear and is the subject of ongoing investigation. Cartoon representations of the neuron (green)–astrocyte (red) interaction in beneficial–supportive (left) versus detrimental–harmful (right) reactive state. Images were initially generated using DALL-E and subsequently refined in Adobe Illustrator to adjust cell shapes, expressions and colors. Note: neurotoxic astrocytes constitute a subset of severe reactive astrocytes, defined by their inflammatory induction and neurotoxic functions, whereas not all severely reactive astrocytes exhibit such detrimental effects Escartin et al. 2021^42^.

The reversibility of astrocyte reactivity depends on the severity, the duration of the insult and the surrounding microenvironment (Fig. 3). In case of mild injuries such as mild trauma or infection, astrocytes can return to their baseline homeostatic state once the stimulus is resolved. For example, reactive astrocytes in the optic nerve can revert to their quiescent state upon the removal of stressors^43^. However, in conditions such as AD or PD, where activation is prolonged or chronic, astrocytes may undergo irreversible changes, contributing to neuroinflammation and scarring through the non-cell-autonomous pathways^20^. Astrocytes sense extracellular changes through receptors such as pattern recognition receptors and cytokine receptors, detecting pathogens, Aβ aggregates or inflammatory mediators^4,41^. Activation involves signaling pathways such as JAK–STAT, NF-κB, mitogen-activated protein kinase (MAPK) and calcineurin, leading to cytoskeletal changes marked by the upregulation of GFAP, vimentin and nestin^35,44,45^.

Reactive astrocytes contribute to both protective and harmful effects. Reactive astrocytes protect the CNS by releasing neurotrophic factors (for example, NGF, TGF-β, LIF and BDNF) and cytokines that promote neuronal survival and create an anti-inflammatory environment^46,47^. Notably, reactive astrocytes closely interact with Aβ plaques and degrade Aβ plaques in an apolipoprotein E epsilon 4 (APOE) gene-dependent manner by releasing amyloid-degrading enzymes such as insulin-degrading enzyme (IDE) and matrix metalloproteinases (MMP-2, MMP-9)^48,49^. They are also involved in the unfolded protein response and immunoproteasome-mediated clearance of misfolded proteins^50^. Furthermore, reactive astrocytes modulate acute neuroinflammation and repair the BBB in a CNS injury model, where their elimination resulted in impaired recovery. Reactive astrocytes can support synaptic plasticity by modulating STAT3 signaling following motor neuron injury. In an ischemic stroke mouse model, reactive astrocytes were linked to vascular repair, as they increased the expression of genes related to angiogenesis. Their chemogenetic ablation led to the impairment of the vascular matrix, disrupted blood flow and worsened motor recovery^51^.

Despite the beneficial effects of reactive astrocytes, there is growing evidence that they adopt a detrimental profile under chronic stress. The release of MMP enzymes by severe reactive astrocytes can impair BBB integrity and trigger the production of proinflammatory cytokines^52^. Neurotoxic reactive astrocytes in specific brain regions are associated with neurodegeneration in AD, PD, MS and Huntington’s disease (HD)^53^. These astrocytes release toxins that damage neurons and oligodendrocytes. For instance, motor neurons were killed by neurotoxins released by astrocytes in a SOD1 mutant mouse model of ALS. Another significant contribution of reactive astrocytes to AD pathogenesis is their involvement in either Aβ clearance or Aβ plaque formation. They have been shown to enhance the expression of the amyloid precursor protein (APP) processing enzyme beta-site APP cleaving enzyme 1 (BACE), thereby promoting Aβ accumulation in patients with AD and various mouse models^54^. Several studies have demonstrated the inhibitory effects of reactive astrocytes on CNS regeneration; for example, the ablation of GFAP or vimentin improved spinal cord recovery after injury^55^. In patients with AD, an increase in astrocyte-derived circulating exosomes has been observed, which transport proinflammatory cytokines, supporting the idea that reactive astrocytes contribute to inflammation^56^. Additional research, including studies from our collaborative research groups, has shown that severe reactive astrocytes exhibit the increased production of H_2_O_2_ through MAO-B, leading to AD pathology hallmarks such as tauopathy, cognitive decline and neuronal atrophy^57^. Our collaborative work demonstrated that severe reactive astrocytes release excessive amounts of the inhibitory gliotransmitter GABA via bestrophin 1 (BEST1) channels, contributing to memory deficits and impairing synaptic plasticity^58,59^. Furthermore, our collaborators also identified an altered metabolic pathway in reactive astrocytes, where the urea cycle leads to enhanced GABA production, further impacting memory function^59^. By contrast, reactive astrocytes modulate Ca^2+^ signaling by overexpressing calcineurin, a Ca^2+^-dependent phosphatase that activates inflammation-related genes. This pathway has been shown to contribute to synaptic dysfunction, neuroinflammation and glutamate dysregulation^60^.

The diverse and dynamic phenotypes of reactive astrocyte reflect their context-dependent heterogeneity and adaptive capacity, which enables structural, molecular and functional adaptations to shifting physiological conditions and pathological insults. This functional flexibility allows astrocytes to support neuroprotection and tissue repair in acute settings; however, persistent or dysregulated activation can drive neurodegeneration through inflammatory signaling, metabolic imbalance and synaptic disruption. Such phenotypic heterogeneity—shaped by both intrinsic programs and extrinsic cues—underscores the limitations of traditional binary models and highlights the need for precise classification systems. Therefore, defining astrocyte subpopulations necessitates a multidimensional framework that considers spatial distribution, morphological features, transcriptional programs, specialized cellular functions and their contributions to disease-associated processes. Recent single-cell and single-nucleus transcriptomic studies have already delineated more than nine transcriptionally distinct astrocyte clusters, underscoring both the complexity of astrocytic heterogeneity and the inadequacy of traditional binary classification schemes^20,61^. However, current resources remain fragmented, disease-specific and often restricted to a single modality. To overcome these limitations, the field increasingly recognizes the need for centralized, multimodal databases that integrate transcriptomic, proteomic, imaging and spatial datasets across neurological conditions. Such efforts would enable the establishment of standardized definitions of reactive subtypes, facilitate cross-disease comparisons and foster cross-laboratory collaboration. Recent large-scale single-cell transcriptomic atlases have already demonstrated the feasibility of systematically classifying glial cell diversity and revealed distinct astrocytes states across disease contexts^62–64^. Ultimately, elucidating the mechanisms that govern transitions between protective and pathological states will be critical to refine classification systems and to guide therapeutic strategies targeting astrocyte reactivity.

## Differences of metabolic function between normal and reactive astrocytes

### Astrocytes as metabolic supporters of neurons in normal conditions

Beyond their physiological roles, astrocytes are key regulators of metabolic processes in the brain. Although the human brain constitutes only 2% of total body weight, it consumes approximately 20% of the body’s oxygen and 25% of its glucose, with even higher demands observed during early development^65^. This substantial energy requirement arises from continuous ion fluxes and other signaling events essential for neuronal function. Given the brain’s dependence on a constant energy supply, any disruption in glucose or oxygen transport can lead to rapid neuronal dysfunction and cell death.

Glucose is fundamental for cellular homeostasis and neuronal migration, and astrocytic glucose metabolism and uptake pathways have been extensively investigated^65^. Unlike neurons, which primarily generate ATP through oxidative phosphorylation, astrocytes predominantly rely on glycolysis for energy production. Moreover, neurons obtain glucose via the pentose phosphate pathway, emphasizing the complementary metabolic roles of these cell types. Alterations in glucose metabolism are early indicators of neurodegenerative disorders, with glucose hypometabolism being a hallmark of AD. The uptake of glucose in astrocytes is modulated by various proteins and ions, including Ca²⁺ and insulin^66^. Insulin plays a neuroprotective role in AD by enhancing Aβ clearance through the upregulation of neprilysin (NEP) and IDE while also stimulating gluconeogenesis following ischemic stroke^67^.

Astrocytes also function as glycogen reservoirs, storing glucose and mobilizing it in response to increased neuronal activity^68^. Under normal glucose conditions, astrocytes continuously generate pyruvate via glycolysis, with excess glucose stored as glycogen. However, excessive glucose concentrations can be detrimental, as demonstrated in studies showing metabolic stress responses in astrocytes exposed to high glucose levels. When glucose availability is limited or neuronal activity is heightened, astrocytes enhance metabolic plasticity by upregulating genes for alternative energy pathways or utilizing lactate and fatty acids as energy substrates^69^. Notably, lactate production and export to neurons is a key astrocytic function, as lactate supports axonal survival even in oxygen-rich conditions. The astrocyte–neuron lactate shuttle (ANLS), first described by Magistretti et al., mediates lactate transfer, and disruptions in this pathway are associated with impairments in neuronal function and memory^70^. Moreover, astrocytes contribute to lipid and ketone metabolism, playing a role in fatty acid oxidation, which supplies up to 20% of the adult brain’s energy needs^35^.

Another essential metabolic function of astrocytes involves the regulation of GABA and glutathione metabolism. Astrocytes and neurons engage in a metabolic exchange via the glutamate–glutamine cycle, which is critical for synaptic function. Within astrocytes, glutamate is converted into glutamine by glutamine synthetase (GS)—an enzyme exclusive to astrocytes in the brain. The resulting glutamine is subsequently transported to neurons, where it is converted back into glutamate by glutaminase, providing a continuous supply of this key excitatory neurotransmitter^35,71^.

Astrocytes regulate synaptic activity by modulating neurotransmitter metabolism and uptake from the synaptic cleft while releasing gliotransmitters into the extracellular space through distinct pathways. Glutamate taken up by astrocytes can be metabolized into glutamine or glutathione, oxidized intracellularly or subsequently released back into the extracellular milieu in a regulated manner. Similarly, GABA, the primary inhibitory neurotransmitter, undergoes oxidation within astrocytes, contributing to ATP production. Moreover, astrocytes participate in de novo glutathione synthesis, thereby playing a crucial role in protecting neurons against oxidative stress and maintaining redox homeostasis^72^. Through these diverse metabolic pathways, astrocytes serve as indispensable metabolic supporters of neurons, ensuring energy balance, neurotransmitter recycling and neuroprotection within the CNS.

### Reactive astrocytes switch their metabolism under disease conditions

Astrocyte metabolism undergoes significant alterations during aging and neurodegenerative diseases, contributing to disease progression and exacerbating neuronal dysfunction. Metabolic changes in astrocytes have been observed even before the clinical onset of neurodegenerative disorders, with glucose metabolism dysregulation and Aβ accumulation being among the earliest pathological hallmarks. Interestingly, the regions affected by metabolic impairments differ between normal aging and AD; in healthy aging, glucose metabolism is primarily reduced in the frontal cortex, whereas in AD, metabolic deficits are more pronounced in the parietal lobe and precuneus^73^. Region-specific neuronal toxicity has been linked to metabolic reprogramming in astrocytes, particularly in response to chronic stress and disease pathology^74^. In their reactive state, astrocytes undergo morphologic and functional remodeling, which alters their ability to provide synaptic and metabolic support to neurons^18,35^. A notable example is the downregulation of GLUT1 glucose transporters, leading to glucose hypometabolism—one of the defining features of AD-related astrocyte dysfunction^75^. In addition, astrocytic connexin channels, which regulate the transport of adenosine triphosphate (ATP), glutamine and glucose, exhibit altered expression patterns in disease states, shifting astrocytes toward either a neuroprotective or neurotoxic metabolic profile^76^.

When exposed to Aβ or other stressors, reactive astrocytes demonstrate increased glucose uptake and glycolysis, leading to enhanced ATP and lactate production^77^. Initially, this metabolic shift may serve as a compensatory mechanism, promoting neuronal energy supply. However, prolonged reliance on glycolysis and subsequent oxidative stress can disrupt mitochondrial function, ultimately accelerating neurodegeneration. In an ischemic mouse model, lactate was shown to trigger astrogliosis through the activation of Akt and STAT3 signaling pathways, further linking metabolic shifts to astrocyte reactivity^78^. In addition, genetic factors such as the APOE genotype influence not only astrocyte metabolism but also their propensity to become reactive. The AD-associated APOE4 allele has been reported to enhance lactate synthesis, thereby altering the metabolic milieu, and to sensitize astrocytes to inflammatory stimuli without overtly changing baseline transcriptomic profiles. Beyond APOE4, other genetic variants directly modulate astrocytic reactivity thresholds. For example, human induced pluripotent stem cell (iPS cell) astrocyte studies indicate that PSEN1 mutations enhance cytokine signaling via disrupted intramembrane proteolysis, lowering the threshold for astrocyte reactivity and predisposing cells to exaggerated inflammatory responses to modest stimuli^79^. In ALS models, SOD1 mutations do not markedly alter astrocytic baseline states but lower the threshold for reactivity, which sensitizes astrocytes to oxidative, glutamatergic and inflammatory stress and drives exaggerated reactive responses to otherwise moderate insults^80^. Collectively, these findings suggest that disease-associated mutations, though mechanistically diverse, converge in their impact on astrocytic reactivity by resetting the threshold at which astrocytes enter reactive states. This shift amplifies maladaptive responses under pathological conditions and reshapes both the metabolic and inflammatory landscape in neurodegenerative disease^81^.

Insulin signaling also plays a critical role in astrocyte metabolism under pathological conditions. Insulin has been shown to enhance the expression of Aβ degrading enzymes in cultured astrocytes, whereas insulin-like growth factor receptor (IGFR) deficiency leads to impaired glucose metabolism and reduced Aβ clearance^56,67^. Beyond glucose metabolism, lipid dysregulation in astrocytes has been implicated in both HD and AD. Although astrocytes can utilize fatty acids as an alternative energy source, prolonged reliance on lipid oxidation results in oxidative stress and mitochondrial impairment, exacerbating neurodegeneration^82^. Glutamine metabolism is significantly altered in reactive astrocytes during AD. Metabolomic analyses of reactive astrocytes in mouse models have revealed that late-stage AD is associated with synaptic dysfunction and neurotransmitter imbalances, primarily due to impaired glutamine synthesis^71^. A reduction in astrocytic glutamine production leads to decreased neuronal GABA synthesis, further contributing to excitotoxicity and synaptic failure.

The primary role of astrocytes is to provide metabolic support to neurons under both physiological and pathological conditions. Their transition to a reactive state is characterized by profound metabolic alterations, marking a shift from a neuroprotective to a potentially neurotoxic profile. This metabolic transformation can be likened to changes observed in the human body during illness, where dietary preferences shift, and metabolic processes are reprogrammed to adapt to stress. However, just as a well-functioning metabolism is essential for sustaining life, the proper metabolic function of astrocytes is crucial for maintaining neural homeostasis. In their healthy state, astrocytes remain indispensable for neuronal support, but their metabolic roles in both health and disease continue to be an active area of research with significant therapeutic potential. Notably, one promising avenue involves leveraging the astrocytic urea cycle to enhance neuroprotection and facilitate a liver-like clearance mechanism in the brain, offering potential strategies for mitigating neurodegenerative processes^59,73^.

## Multidimensional roles of astrocytes in coping with oxidative stress

### How do healthy astrocytes protect neurons from ROS/reactive nitrogen species?

The brain utilizes approximately 20% of the body’s total energy and is highly susceptible to oxidative stress owing to its high content of polyunsaturated fatty acids, which are prone to oxidation. Moreover, glucose metabolism through cellular respiration generates reactive oxygen species (ROS) and reactive nitrogen species, which can cause lipid peroxidation, DNA damage and protein degradation, ultimately leading to neuronal death. Neurons are particularly vulnerable to oxidative stress because of their high oxygen demand, abundance of unsaturated lipids and relatively low antioxidant capacity^83^. Consequently, neuronal survival depends heavily on the antioxidative functions of astrocytes, which engage in close metabolic crosstalk with neurons.

Oxidative stress can arise from various sources, including mitochondrial dysfunction, impaired autophagy, lipid peroxidation, misfolded proteins and cytosolic ROS-producing enzymes. Astrocytes serve as neuroprotectors by mitigating oxidative damage through intrinsic antioxidative pathways, mediated via the Nrf2 signaling pathway^84^. However, under pathological conditions, astrocytes change their antioxidant capabilities. Recent studies have identified several key regulators of astrocytic antioxidant functions, including the mGLUT3 receptor, cyclic AMP, ROS-induced mitochondrial DNA release and dystrophin^85,86^. Astrocytes maintain their antioxidative functions through two primary mechanisms: the Nrf2–keap1–antioxidant response element (ARE) pathway and glutathione (GSH) synthesis. Nrf2, a transcription factor, is activated upon oxidative stress signals and binds to the ARE in the promoter regions of target genes, triggering the expression of antioxidative enzymes^83^. Notably, astrocytes exhibit high Nrf2 activity, whereas neurons rely on astrocytic Nrf2-mediated protection. Furthermore, astrocyte-derived H₂O₂ has been shown to activate Nrf2, reinforcing antioxidative responses^87^.

Astrocytes synthesize and store GSH, releasing it into the extracellular environment to protect neurons from oxidative damage. GSH production is regulated by inflammation-associated pathways, and the disruption of STAT3 signaling reduces GSH levels, leading to an increased accumulation of ROS. In addition, IL-1β, a cytokine known to enhance oxidative stress in the CNS, has been found to exert protective effects on both astrocytes and neurons by modulating GSH synthesis^88^. Beyond their intrinsic antioxidative mechanisms, astrocytes can also transfer functional mitochondria to neighboring cells. In an in vitro neuron culture model of intracerebral hemorrhage, the transplantation of astrocytic mitochondria restored the function of the damaged Mn-SOD enzyme^89^. These findings highlight the role of astrocytes in maintaining redox balance in the brain not only through direct antioxidant mechanisms but also through intercellular metabolic support.

### Astrocytes become frenemies to neurons when stress levels exceed the threshold

Astrocytes take place in antioxidative defense in the CNS, but when oxidative stress surpasses their regulatory capacity, they undergo pathological changes that exacerbate neuronal damage (Fig. 3) ^83^. Once astrocytes become reactive, their ability to mitigate oxidative stress diminishes, and they may actively contribute to redox imbalance. Their heterogeneous reactivity profile can result in either proinflammatory or anti-inflammatory traits, altering the delicate balance of brain homeostasis^18^. Proinflammatory cytokines play a key role in astrocytic transformation, promoting increased APP expression, β-site APP cleaving enzyme 1 activity and secreted Aβ levels, all of which are linked to neurodegenerative disease pathology^90^.

Mitochondria, the primary organelles responsible for maintaining redox balance in astrocytes, are not only concentrated in the cell body but also found near synapses, where they regulate oxidative stress. Mitochondrial dysfunction in astrocytes has been associated with the progression of neurodegenerative diseases such as ALS and PD, contributing to motor neuron degeneration. Studies indicate that reducing mitochondrial ROS levels can improve cell-specific cognitive function, highlighting the direct impact of astrocyte-derived oxidative stress on neuronal survival^91,92^. Moreover, IGF-1 signaling, which is implicated in neurodegeneration, modulates astrocytic redox balance by modifying mitochondrial activity^82^.

Reactive astrocytes, in response to oxidative stress, alter the expression of key enzymes such as nitric oxide synthase (NOS), NADPH oxidase (NOX) enzymes and fatty acid amide hydrolase, which, in turn, enhance the production of oxidative agents^93^. NOX activity increases with aging, leading to excessive superoxide production and mitochondrial impairment, which worsens disease pathology^83^. In AD models, NOX2 upregulation following Aβ stimulation promotes astrocyte reactivity, whereas NOX4 overexpression triggers oxidative stress-induced lipid peroxidation and mitochondrial dysfunction^94^. In PD models, interventions targeting NOX enzymes, such as curcumin treatment, have been explored as potential neuroprotective strategies^95^.

The Nrf2 pathway, a major antioxidative mechanism in astrocytes, also becomes dysregulated during neurodegeneration. In PD, elevated dopamine levels can enhance ROS production, whereas GSK-3β, a key regulator of tau hyperphosphorylation in AD, accelerates Nrf2 degradation, impairing the astrocytic defense system^87^. The involvement of reactive astrocytes in oxidative stress and their contribution to AD has been widely studied. Evidence suggests that oxidative stress is already present in the early stages of AD, contributing to disease progression^96^. The accumulation of Aβ proteins is closely associated with increased oxidative products, further amplifying cellular damage^90^. Beyond AD, astrocyte-mediated ROS homeostasis disruption has been implicated in the neurodevelopmental disorder Fragile X syndrome. Studies using the *Fmr1* KO mouse model demonstrated that reactive astrocytes exacerbate oxidative stress by increasing ROS emission^97^.

In this context, the oxidative stress response of both healthy and reactive astrocytes is evaluated. Healthy astrocytes play a vital role in maintaining redox balance, mitigating oxidative damage and supporting neuronal survival in the highly oxidative environment of the brain. As part of their defense mechanism, they release inflammatory cytokines to recruit immune cells, aiming to restore homeostasis. However, when oxidative stress surpasses their regulatory capacity, astrocytes transition into a reactive state, leading to excessive ROS production and neuroinflammation. Although initially serving a protective role, reactive astrocytes can inadvertently exacerbate oxidative damage, disrupt neuronal function and contribute to neurodegenerative disease progression. Although compensatory mechanisms attempt to counteract these harmful effects, prolonged astrocyte activation often leads to irreversible pathological conditions, tipping the balance toward neuronal degeneration (Fig. 3). Ultimately, under oxidative stress, healthy astrocytes remain the primary protectors of neuronal integrity, ensuring cellular survival in one of the body’s most oxidative environments—the brain.

## Dynamic ion signaling in astrocytes

### Astrocytes as the signal powerhouse of the brain

**Fig. 4:**
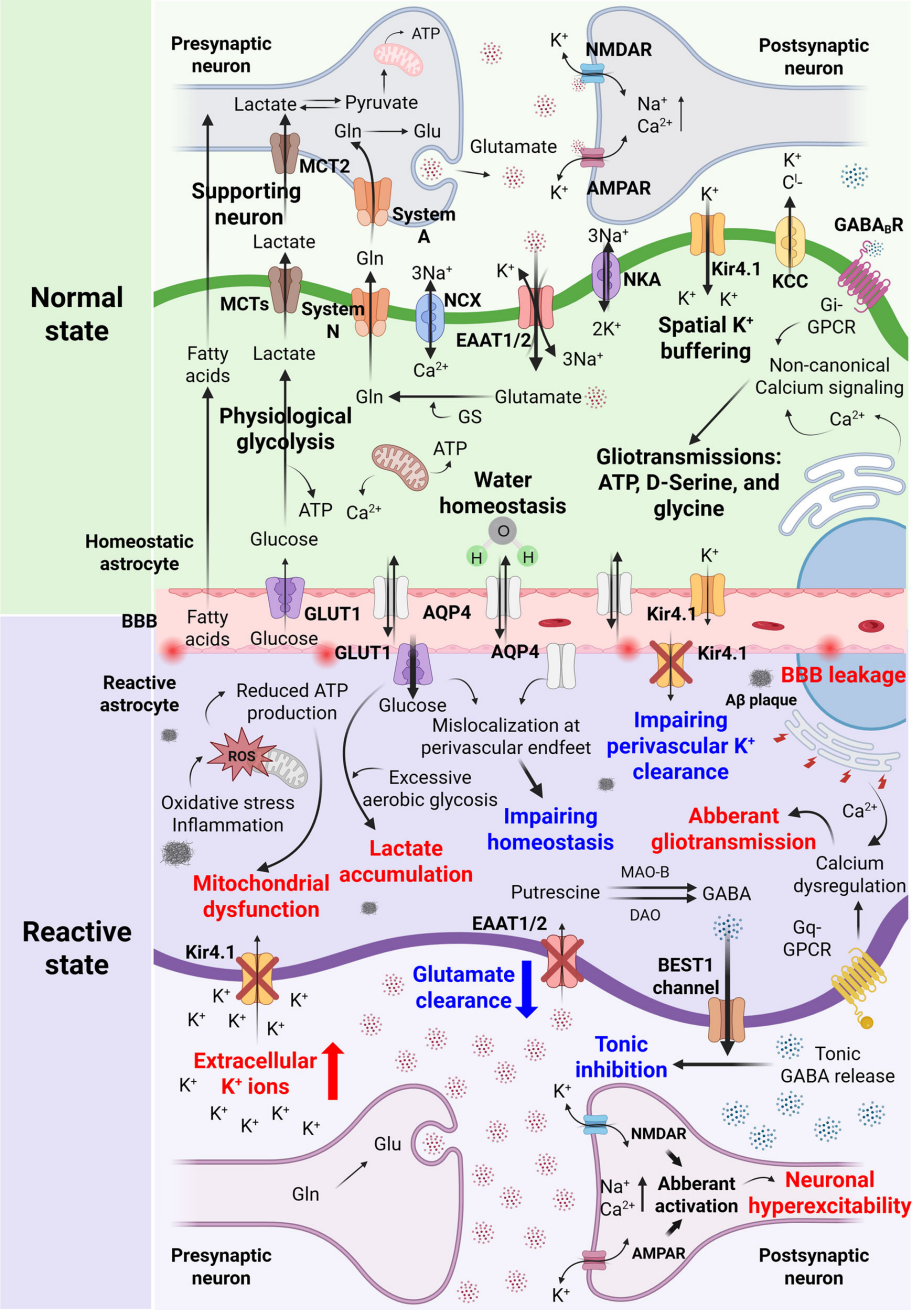
Astrocyte functions at the tripartite synapse differ between physiological (normal) conditions and reactive disease conditions. Top, green: normal astrocytic functions that support neuronal activity, including glutamate uptake, lactate shuttling, K^+^ buffering, gliotransmitter release and the maintenance of water and metabolic homeostasis. Bottom, purple: reactive astrocytic alteration, including mitochondrial dysfunction, excessive lactate accumulation, impaired perivascular K^+^ clearance, BBB leakage, aberrant gliotransmission and dysregulated Ca^2+^ signaling. Blue labels and arrows denote homeostatic astrocytic functions, whereas red labels and arrows denote pathological alterations observed in the reactive state. At the center, the perivascular interface between astrocytes and the BBB is depicted, highlighting their anatomical positioning and functional specialization in maintaining vascular-synaptic communication and homeostasis.

As the central interface between neurons and the extracellular environment, astrocytes orchestrate multiple ion transport mechanisms in the CNS. As with previous topics, this function plays a critical role in responding to external stimuli, inflammation and infections by altering the open/close states of ion channels or modulating the expression of corresponding proteins, as shown in Fig. 4. Their crosstalk with neurons through ion signaling supports synapse formation during neurodevelopment. Astrocytes rely on ions to generate excitatory signals, compensating for their lack of electrical excitability^98^. A major aspect of ion regulation in astrocytes is Ca²⁺ signaling. Ca²⁺ can originate from intracellular organelles or enter from the extracellular environment through specific ion channels, including Ca²⁺ release-activated Ca²⁺ channels, transient receptor potential (TRP) channels and L-type voltage-gated Ca²⁺ channels. This Ca²⁺ signaling influences neuronal activity and behavior by participating in bidirectional neuron–glial communication. It also modulates the release of glutamate, D-serine and ATP from astrocytes^99^. The regulation of Ca²⁺ signaling in astrocytes is primarily mediated by inositol trisphosphate receptors (IP3Rs), which have been studied for their potential roles in brain damage and neuronal death in mouse models^100^. Furthermore, disruptions in Ca²⁺ signaling can lead to abnormal behaviors, such as excessive self-grooming, a process influenced by GABA receptors^101^.

Sodium (Na⁺) ion transport is another essential function of astrocytes, contributing to the movement of neurotransmitters, ions and metabolites across the astrocytic membrane. Key Na⁺ transporters and channels found in astrocytes include the Na^+^/K^+^ pump, ionotropic receptors such as AMPA and NMDA receptors, TRP1/4/5 channels and GABA and glycine transporters. Na⁺ signaling can modulate the activity of these transporters, sometimes causing them to operate in reverse. This reversal can lead to inhibitory effects on neuronal activity through mechanisms involving the Na⁺/Ca^2^⁺ exchanger (NCX), as well as GABA and glycine transporters^99,102^. Astrocytes regulate their energy metabolism and can respond rapidly to environmental changes with the help of Na^+^ oscillations. Na⁺ influx has been shown to trigger glucose and lactate metabolism, further emphasizing the importance of Na⁺ in astrocytic energy regulation^103^.

In addition to Na⁺ and Ca²⁺, astrocytes regulate the K^+^ transport of ion exchange processes. Astrocytic K^+^ channels and transporters facilitate the spatial buffering of extracellular K^+^ by internalizing excess K^+^ in regions of heightened neuronal activity and redistributing it toward less active areas via gap junction-coupled astrocytic networks. K^+^ efflux and influx occur through the Na^+^/K^+^ pump, K^+^/Cl^−^ co-transporter, Na^+^/K^+^/Cl^−^ co-transporter and the Kir4.1 K^+^ channel^77^. Through the Na^+^/K^+^ pump, astrocytes accumulate K^+^ during periods of high neuronal activity and release it back to neurons when activity subsides. Increased extracellular and intracellular K^+^ concentrations have been shown to affect glycolysis through Na^+^/K^+^-ATPase and pyruvate carboxylase, respectively^104^. In addition to ion regulation, astrocytes also facilitate the transport of vitamin E to neighboring neurons, supporting brain health and cognitive function^105^.

Under physiological conditions, astrocytes support synaptic transmission by efficiently clearing glutamate via excitatory amino acid transporters (EAAT)1/2^106^, converting it into glutamine and supplying lactate to neurons through aerobic glycolysis^107^. K^+^ balance and water homeostasis are maintained through Kir4.1 and AQP4 channels, respectively, which are localized at the perivascular endfeet^108,109^. In addition, astrocytic glucose uptake via GLUT1 fuels glycolytic pathways to support neuronal activity^110,111^.

### Reactive astrocytes undergo changes in their signaling pathways under pathological conditions

Reactive astrocytes exhibit pathological changes, including enhanced glycolytic flux and excessive lactate accumulation. The mislocalization or downregulation of Kir4.1 and AQP4 disrupts K^+^ clearance and water homeostasis^112,113^. Impaired glutamate uptake leads to extracellular accumulation, triggering the overactivation of AMPA and NMDA receptors and driving neuronal hyperexcitability. In parallel, Ca^2+^ dysregulation, endoplasmic reticulum stress and mitochondrial dysfunction further exacerbate astrocytic impairment^114^. Notably, reactive astrocytes aberrantly produce GABA from putrescine via MAO-B, releasing it through the BEST1 channel^58,59^.

Similar to healthy astrocytes, reactive astrocytes also rely on Ca^2+^ signaling under disease conditions^115^. Extensive studies have shown that exposure to Aβ triggers Ca^2+^ signaling in astrocytes via α7 receptors, whereas IP3 receptors—particularly IP3R1 and IP3R3—further modulate this signaling pathway. Studies using IP3R2 knockout models have shown that the absence of this receptor in astrocytes reduces neuronal death and astrocyte reactivity following ischemic shock^100,116^. In addition, Orai1, a Ca^2+^ channel, has emerged as a key regulator of reactive astrocytes by controlling the synthesis and release of a broad range of proinflammatory mediators^113^.

Beyond their role in ion regulation through membrane channels, astrocytes also secrete small extracellular vesicles that contribute to recovery after spinal cord injury and promote synapse formation via fibulin-2–TGF-β signaling. When the CNS undergoes insult or injury, reactive astrocytes enhance communication with other cell types to manage inflammatory responses and support neuronal recovery. One study demonstrated that reactive astrocytes alter their secretome profile following an inflammatory insult, with 149 proteins showing differential expression—including components of the renin–angiotensin system, a critical part of the signaling network^117^. Moreover, reactive astrocytes can modify their membrane channel expression in disease states, as exemplified by altered dopamine D2 receptors expression in HD^118^.

Along with the remodeling of intracellular signaling pathways, reactive astrocytes secrete a variety of neurotoxic and immunomodulatory molecules that profoundly influence intercellular communication and immune responses within the brain. Key astrocyte-derived factors include C-X-C motif chemokine ligand (CXCL10), stromal-derived factor 1 (CXCL12), Fas ligand (FasL/CD95L), TNF superfamily member 10, TNF-related apoptosis-inducing ligand 2 (TRAIL), high mobility group B1 protein (HMGB1), Aβ and lipocalin-2 (LCN-2). In the case of ischemic stroke, reactive astrocytes also contribute to BBB permeability through the Wnt/β-catenin pathway as demonstrated in an Nhe1 conditional knockout mouse model^119^. The role of the Wnt pathway in both reactive and nonreactive state of astrocytes has been examined in various studies, with evidence suggesting that it influences astrocytes communication with other cells in the CNS by stimulating the transcription of synaptogenic factors^120^.

**Fig. 5:**
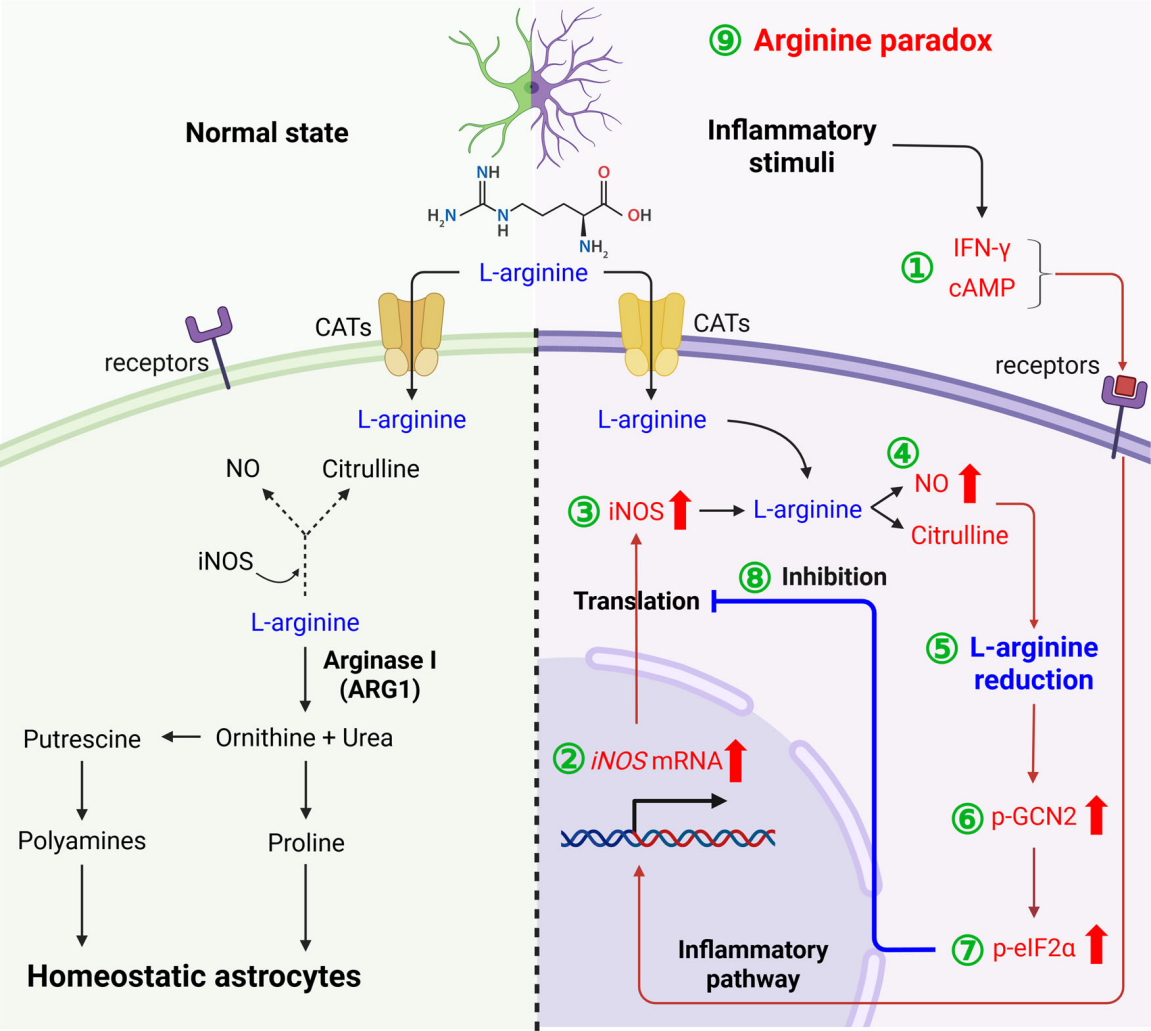
Schematic representation of the arginine paradox in astrocytes under normal and inflammatory conditions. Left: under physiological conditions, astrocytes exhibit basal expression of cationic amino acid transporters (CATs), and L-arginine uptake supports minimal NO production owing to the absence of iNOS expression. Right: by contrast, inflammatory stimuli such as IFN-γ and cAMP (1) induce robust iNOS mRNA expression (2) and promote iNOS protein synthesis (3). Elevated iNOS level rapidly depletes intracellular L-arginine (4), resulting in increased NO and citrulline production. The consequent reduction in arginine (5) activates the general control nonderepressible 2 (GCN2) (6), which phosphorylates eIF2α (7), leading to translational inhibition of iNOS (8). However, increased CAT expression under inflammatory conditions restores intracellular arginine levels, alleviating translational repression and restoring NO synthesis—highlighting the ‘arginine paradox’, wherein extracellular arginine regulates NO production despite intracellular arginine sufficiency (9). This process illustrates the arginine paradox, wherein extracellular L-arginine supplementation enhances NO production despite apparently sufficient intracellular arginine levels. Thus, extracellular arginine can override translational repression, amplifying NO synthesis in reactive astrocytes. Blue labels/arrows denote homeostatic astrocytic functions, red labels/arrows denote pathological or inflammatory changes and green numbers denote key steps in the signaling cascade.

Beyond these transcriptional and secretory adaptations, previous studies have highlighted that astrocytic inflammatory responses are also tightly regulated at the translational level^121,122^ (Fig. 5). L-arginine, the sole endogenous nitrogen donor for NOS, governs nitric oxide (NO) production during nervous system development and in various pathological conditions, including stroke, multiple sclerosis, PD and HIV-associated dementia. Lee et al. demonstrated that L-arginine availability regulates the translation of inducible NOS (iNOS) without altering its transcription or mRNA stability. In cytokine-stimulated astrocytes, arginine deprivation activates the amino acid-sensing kinase GCN2, leading to phosphorylation of eukaryotic initiation factor 2α (eIF2α) and suppression of iNOS protein synthesis, thereby attenuating NO production. This regulatory mechanism explains the so-called ‘arginine paradox’, which refers to the phenomenon where extracellular L-arginine availability influences NO production despite the saturation of NOS enzymes with intracellular arginine. These findings unveil a distinct regulatory axis in which nutrient sensing by astrocytes fine-tunes inflammatory and oxidative responses at the level of protein translation, revealing a novel mechanism by which substrate availability modulates enzyme activity during neuroinflammation^123^.

Intracellular and intercellular signaling pathways are fundamental to nervous system homeostasis, and disruptions in these processes can lead to serious consequences. As key regulators of ion and metabolite transport between neurons and other glial cells, astrocytes serve as essential hubs in the brain’s network. Their reactivity represents a direct adaptation to environmental cues, and the differences in signal exchange between reactive and nonreactive astrocytes are vital components of the brain’s immune defense. Taken together, these observations underscore how astrocytic metabolic and transporter dysfunctions underlie synaptic dysregulation and BBB disturbances in neurodegenerative diseases.

## Exploring the potential of astrocytes as therapeutic targets for neurodegenerative disorders

### What therapeutic benefits can healthy astrocytes offer in AD?

As emphasized throughout this review, astrocytes play fundamental roles in the CNS and neurodegeneration. Beyond their supportive functions for the brain, they also hold significant potential as therapeutic target under neurodegenerative conditions^124^. Whereas astrocytes may change their roles during disease progression, both their healthy and reactive states present opportunities for targeted treatment. Various studies have explored ways to harness healthy astrocytes for neurodegenerative disease therapies, focusing on enhancing their metabolic functions, restoring normal receptor and transporter activity or utilizing their neuroprotective properties^125^.

One such approach involves increasing the expression of EAATs to improve glutamate metabolism, thereby supporting neuronal survival. For instance, the beta-lactam antibiotic ceftriaxone has shown to elevate EAAT transport levels in astrocytes, leading to glutamate clearance and neuronal protection^126^. Moreover, astrocyte-derived neurotrophic factors are considered valuable targets for AD and ischemic stroke, as they stimulate astrocyte-mediated neurogenesis and angiogenesis or enhance their neuroprotective profiles through growth factors and antioxidants^124,127^. Bilobalide, a compound extracted from *Ginkgo biloba*, has shown neuroprotective effects and was investigated in an in vivo AD model by Xiang and colleagues^128^. Their findings indicate that bilobalide enhance astrocytic Aβ clearance by upregulating Aβ-degrading enzyme expression. In addition, several phytochemicals, including curcumin, neurosteroids and phytoestrogens, have been shown to improve antioxidative function of astrocytes^129^. These therapeutic approaches could also be extended to other neurodegenerative conditions affecting the CNS.

Another promising strategy involves astrocyte transplantation to restore brain function^89^. Compared with neuronal transplantation, transplanted astrocytes better integrate into the local cellular network, allowing them to replace dysfunctional astrocytes and reinstitute homeostasis. This method has been explored in AD, PD and HD, despite their differing etiologies. For example, one study utilized enteric glial cells as an astrocyte source and examined the improvement in neuropathology and cognitive function following transplantation^130^. Similarly, targeting astrocytes for ALS treatment has shown promise. Transplanting embryonic stem cell-derived astrocytes into murine ALS models with the SODG93A mutation has been found to restore young astrocyte characteristics, potentially slowing disease progression^131^.

Given that neurons have limited regenerative capacity, reprogramming astrocytes into neurons could serve as an alternative solution. Several studies have shown that astrocytes can be genetically reprogrammed to adopt neuronal identities and restore neural circuitry^25^. Interestingly, miRNA-based approaches have also been used to induce astrocyte-to-neuron conversion, offering the potential to recover cognitive function. In particular, miR-132 has been shown to promote neuronal survival and morphogenesis by targeting IL-1 receptor-associated kinase, thereby suppressing astrocyte-derived proinflammatory cytokine secretion and fostering a neuroprotective environment^132^.

### How can reactive astrocytes be a therapeutic target in AD?

Reactive astrocytes have emerged as significant players in AD, as they can promote neurodegeneration. Therefore, targeting reactive astrocytes offers a promising avenue for therapeutic strategies^124^. In the transgenic APPswePS1dE9 (APP/PS1) mouse model, which stimulates amyloidosis and gliosis, targeting reactive astrocytes improved cognitive function demonstrating their potential for treatment in AD^133^. Given that reactive astrocytes can adopt either neurotoxic or neuroprotective phenotypes, both profiles present opportunities for interventions. For the neurotoxic profile, reducing GABA synthesis inhibition or preventing the transformation into the toxic state through microglia modulation have demonstrated improvements in cognitive outcomes^58,134^. In addition, targeting proinflammatory cytokines has proven effective in mitigating inflammation and restoring memory performance in animal models^88,127^. Conversely, enhancing the neuroprotective functions of astrocytes may further support neuroprotection and slow neurodegenerative processes. For example, insulin treatment has been shown to improve cognitive function in AD models by activating insulin receptors on these astrocytes^67,82^.

Cytokine-based therapies also hold promise in targeting reactive astrocytes in AD^120^. Tissue inhibitor of metalloproteinase-1 (TIMP-1), a cytokine produced by neuroprotective astrocytes, has been shown to stimulate Aβ clearance and improve cognitive function in AD mice^135^. Similarly, cytokines including IL-10, IL-33 and interferon-β have been studied for their potential to suppress proinflammatory cytokine production and enhance memory^136^. Furthermore, reactive astrocytes secret neurotrophic factors such as Nrf2, which can be leveraged to promote neuroprotection and preserve cognitive function^85,96^.

Targeting specific molecular pathways in reactive astrocytes presents additional therapeutic opportunities. These include inhibiting the JAK2–STAT3 signaling pathway, reducing GFAP expression, blocking inflammatory pathways such as NF-κB, regulating neurotransmitter secretion such as GABA and modulating APOE expression to control astrogliosis^81,137,138^. It is important to emphasize that activation of the JAK–STAT3 pathway and the upregulation of GFAP should not be interpreted as substate-specific markers of astrocyte reactivity^139^. Rather, these signals reflect context-dependent processes that can drive both beneficial and detrimental outcomes, ranging from tissue repair and neuroprotection to inflammation and neurodegeneration. For example, astrocyte-specific expression of SOCS3 in AD mouse models was shown to suppress not only GFAP induction and morphological hypertrophy but also neuroprotective and neurotoxic associated markers while simultaneously improving amyloid burden, synaptic function and cognition. These findings underscore that JAK–STAT3 signaling broadly regulates astrocytic reactivity across contexts, highlighting the need for refined biomarkers to distinguish adaptive from maladaptive states^140^. Although GFAP remains widely used as a clinical marker of astrogliosis, it lacks the specificity required to distinguish maladaptive from adaptive astrocytic states, thereby limiting its diagnostic and prognostic utility. To address this limitation, multimodal biomarkers have been developed, including fluid markers such as plasma GFAP and chitinase-3-like protein 1 (YKL-40) that differentially associate with amyloid versus tau pathology, positron emission tomography (PET) tracers targeting astrocytic MAO-B that provide complementary in vivo readouts and proteomic analyses identifying novel candidates such as VCAM1 and BST2^141–144^. Together, these advances highlight ongoing progress toward defining substate-specific biomarkers that can distinguish harmful from adaptive astrocyte responses to guide therapeutic targeting of astrocyte reactivity in neurological disease.

Given their high plasticity and stem-cell-like characteristics, reactive astrocytes have been investigated as potential therapeutic targets for in neurological disorders such as ischemic stroke. For example, mesenchymal stem cell-derived cytokines have been shown to modulate astrocytic responses, with IL-6 preventing the progression of astrocyte reactivity following ischemic brain injury through the activation of the AMPK–mTOR signaling pathway^145^. Beyond their reactivity in stroke, astrocytes also exhibit a form of ‘autophagy plasticity’, dynamically adjusting autophagic activity in response to pathological stimuli such as Aβ accumulation. This capacity suggests that modulating astrocytic autophagy could enhance protein clearance and cognitive function in AD^13,146^. However, accumulating evidence indicates that autophagy is not universally protective: when chronically or excessively activated, it may become maladaptive. Impaired lysosomal flux or sustained oxidative stress, for instance, can convert autophagy from a protective mechanism into a driver of lysosomal dysfunction, mitochondrial stress and neuronal vulnerability^147,148^. Together, these observations highlight the dual role of astrocytic autophagy—supporting clearance and repair under certain conditions, yet exacerbating pathology when dysregulated. Therefore, therapeutic strategies targeting astrocytic autophagy should be carefully tailored in a disease-stage- and context-dependent manner.

Reactive astrocytes, similar to nonreactive astrocytes, can be reprogrammed into neurons. Research on AD models has suggested that reactive astrocytes are more susceptible to neuronal reprogramming than their nonreactive counterparts, underscoring the role of astrocytic heterogeneity in influencing cell differentiation^25,149^. In an in vivo AD model, reactive astrocytes were converted into neurons via the expression of miR-302/367 cluster. This reprogramming resulted in improved memory function and learning abilities^150^. Several strategies have been explored to target astrocyte reactivity and to mitigate AD progression. Adeno-associated virus was used to target hippocampal astrocytes in an APPP/PS1 mice for inducing VIVIT expression, which inhibits reactivity via the calcineurin pathway. This treatment represses astrocytic reactivity, decreases Aβ load and enhances cognitive function. Furthermore, studies done with acetylcholinesterase inhibitors, which are common AD treatments, showed the reversibility of reactive astrocytes to a healthy state after treatment^151^.

Interestingly, astrocyte reactivity is influenced and modulated by microglia activation, suggesting that drugs targeting microglia may offer an indirect strategy to combat reactive astrocytes. Glucagon-like peptide-1 receptor (GLP-1R) agonist NLY01 prevents the generation of reactivity in astrocytes and protects neurons from cell death^152^. Moreover, the matrine-derived compound MASM, which has anticancer and anti-inflammatory effects, has been shown to suppress the transformation of astrocytes into the toxic form, offering benefits in conditions such as autoimmune encephalomyelitis and multiple sclerosis^153^. Alterations in Ca^2+^ homeostasis due to neurodegenerative diseases affect signal transducers such as TRP channels. This can increase ROS production, inflammation and disrupt mitochondrial function. Research has indicated that compounds targeting TRP channels, such as vanillin, capsaicin and capsazepine, can reduce reactive astrocyte activity, which in turn can mitigate Aβ toxicity in the early stages of AD^154^.

In recent years, astrocyte-targeted therapeutic approaches have expanded from preclinical interventions to early clinical trials. Strategies such as HDAC inhibition, astrocyte transplantation and modulation of autophagy or metabolic pathways remain confined to experimental models, whereas GLP-1 receptor agonists and MAO-B inhibitors have advanced to clinical testing and show effects on astrocytic metabolism and neuroinflammation. At the same time, astrocytic biomarkers such as plasma GFAP are being evaluated as translational tools in ongoing trials. Major barriers to clinical application include limited BBB penetration, subtype heterogeneity and long-term safety of cell-based approaches. Future studies should incorporate human iPS cell-derived astrocyte models, precision delivery platforms and multimodal biomarkers to bridge these gaps and accelerate astrocyte-based therapies from mechanistic insight to clinical practice.

In the final section of this discussion, the therapeutic potential of healthy and reactive astrocytes was examined. As astrocytes play a dual role in both preventing and contributing to disease pathogenesis, they represent an important target in AD research, where various mechanisms can be used to modulate their activity for therapeutic benefit^20,125^.

## Conclusion

Astrocytes are a fundamental cell type in the brain and are involved in supporting neurons through the regulation of metabolic pathways, ion signaling cascades, clearance of toxic molecules and initiation of BBB formation. In response to various brain stresses, astrocytes undergo functional and morphological changes, either signaling the immune system or directly mitigating damage within the brain. Astrogliosis, a common pathological phenotype in neurodegenerative disorders, is characterized by alterations in gene expression—such as the upregulation of GFAP—and distinct morphological changes^4^. There may be a threshold at which the neuroprotective functions of reactive astrocytes shift toward neurotoxic effects, acting as adversaries of neurons. Although astrocyte reactivity serves as an initial defensive mechanism, excessive or prolonged reactivity has been implicated in the progression of neurodegenerative diseases. Once activated, reactive astrocytes can exacerbate inflammation, trigger apoptotic pathways in neurons and contribute to Aβ accumulation. Moreover, astrocyte reactivity itself exhibits heterogeneity, meaning not all reactive astrocytes behave in the same way. Some maintain their protective function, and under conditions of mild or moderate stress, they may even revert from a neurotoxic to a neuroprotective profile^18,155^. Although some threshold that governs the transition of astrocytes into a reactive is thought to exist, the precise point at which this activation occurs—and how astrocytes regulate this shift—remains unclear. Critical questions regarding the molecular mechanisms driving the transformation of healthy astrocytes into their reactive form or vice versa need to be addressed in future study. Another important unanswered question concerns the pathophysiological interactions between astrocytes and other glial cells, such as microglia and oligodendrocytes, under neurodegenerative conditions and their associated microenvironments. Advanced experimental techniques, including spatial transcriptome, single-cell (or nuclei) transcriptomics and assay for transposase-accessible chromatin using sequencing, will be instrumental in uncovering the epigenetic and transcriptomic signatures associated with the transformation of astrocytes under neurodegenerative conditions^156^. Looking ahead, centralized multimodal repositories that integrate transcriptomic, proteomic and imaging data across neurological diseases will be essential to standardize reactive astrocyte definitions and foster cross-laboratory collaboration, ultimately refining subtype classification and accelerating translation. It should be acknowledged that many insights into astrocyte biology have been derived from in vitro models; however, these systems often flatten or artifactually skew the diversity of reactive astrocytic states observed in vivo. Such limitations necessitate cautious interpretation when extrapolating findings from cultured astrocytes to physiological or pathological contexts. Recent methodological advances—including organotypic slice cultures, three-dimensional brain organoids and in vivo imaging approaches—offer scalable and physiologically relevant platforms that more faithfully capture astrocyte heterogeneity and functional consequences. Incorporating these approaches will be essential to refine our understanding of astrocytic diversity and to bridge the gap between experimental models and human disease. In this Review, we compared the distinct characteristics and identities of normal and reactive astrocytes, assessing their dual role as either friends or foes to neurons. It is important to avoid characterizing reactive astrocytes solely as ‘frenemies’ of neurons, as their transformation represents an adaptive mechanism aimed at enhancing survival. The neurotoxic behavior of severely reactive astrocytes under chronic stress conditions should be distinguished from the characteristics of healthy or mildly reactive astrocytes (Fig. 3). Given that the heterogeneity of the reactive astrocyte profile depends on the level and duration of stress exposure, a single therapeutic approach is unlikely to be sufficient for treating multifactorial neurological diseases. In this context, breakthrough research and innovative strategies should be used to develop therapies for astrocyte-mediated neurodegeneration by modulating different levels of astrocyte reactivity.
